# The Efficiency of self-employed general practitioners and factors affecting it: a study in Iran

**DOI:** 10.1186/s13104-020-05104-3

**Published:** 2020-06-01

**Authors:** Razie Hajibagheri, Farhad Lotfi, Mohsen Bayati

**Affiliations:** 1 Student Research Committee, Shiraz University of Medical Sciences, Shiraz, Iran; 2 Health Human Resources Research Center, School of Management & Information Sciences, Shiraz University of Medical Sciences, Shiraz, Iran; 3 Health Human Resources Research Center, School of Management & Information Sciences, Shiraz University of Medical Sciences, Almas Building, Alley 29, Qasrodasht Ave, Shiraz, Iran

**Keywords:** Efficiency, General practitioner, Medical economics, Economic competition, Data envelopment analysis

## Abstract

**Objective:**

Physicians as an economic firm make use of available resources such as time, human forces and space to provide healthcare services. The current study aimed at estimating the technical efficiency of Iranian self-employed general practitioners (GPs) and its effective factors using data envelopment analysis and regression analysis.

**Results:**

About 2% of the GPs were fully efficient and the remaining (98%) were inefficient. Almost, 2.09% of the physicians had constant returns to scale, and 31.41% and 66.49% of them had increasing and decreasing returns to scale, respectively. According to the regression estimates, gender (female) (β = 3.776, P = 0.072), age (β = 0.475, P = 0.013), practice experience (β = − 0.477, P = 0.015), contract with the insurer (β = − 6.475, P = 0.005) and economic expectations (β = 1.939, P = 0.014) showed significant effect on GPs inefficiency. Most of the GPs surveyed did not optimally allocate their time and physical and human resources to provide their services. Female GPs, older ones, those with fewer practice experience, those with higher economic expectations, and the GPs with no insurance contract were more inefficient. Increasing the insurance coverage of self-employed GPs and providing them with training in office economic management can reduce their inefficiency.

## Introduction

One of the most important components of any health system is physician workforces [1], who make vital decisions in the health care system. In fact, as they play the role of agents to direct other medical inputs, their decisions significantly affect the quantity, quality, and costs of the health care services. Therefore, physicians’ behavior is a fundamental issue in health economics, as it will affect other functions and goals of the health system [2].

A general practitioner’s (GP) visit is of great importance because it is often the start point for benefitting from other health services [3, 4]. In low-income countries and for low-income individuals, referral to a GP is one of the cost-effective ways to receive primary care in order to reduce extra costs and unnecessary referrals to specialists and ultimately hospitalization rates [5]. Despite the role of GPs in community health, given the significant impact of physicians on resource orientation as well as the likelihood of induced demands and the large amount of resources to be reimbursed, outputs are not as efficient and of sufficient quality [6].

Considering resource constraints and cost pressures, especially in the social sectors of countries, optimal use of resources and efficiency enhancement are emphasized [7]. Health care is an economic commodity. Patients play the role of consumers, and health care providers are suppliers [8]. Among health care providers, physicians as one of the most important firm provide health services. Like any other firm, physicians make use of available resources such as time, human forces and space to provide healthcare products. There are various theories about the economic behavior of physicians as a firm, including profit maximization model, utility maximization model, and target income hypothesis [9]. Efficiency is a factor on which most firms in today’s world rely to compete [10]. Hence, to evaluate economic performance of physicians, like other firms, it is helpful to examine the concept of efficiency.

Very little research has been done on this issue. A study by Heimeshoff et al. [11] in 2013 examined the costs and technical efficiency of 3126 physicians in Germany from 2006 to 2008. Also, Olsen et al. [12] in Denmark (2011) estimated the production frontier of GPs considering the number of physician visits and a production criterion as outputs. Estimating the physician production function in the US, Reinhardt showed that physicians could produce more than they were doing and increase their productivity [13]. DeFelice and Bradford investigated the inefficiency of individual and group production of 924 GPs [14].

To the knowledge of the researchers, no studies had been done on the efficiency of physicians in developing countries.

GPs in Iran, as the most important providers of primary health care, play a significant role in the overall functioning of the health system. Given the volume and variety of services provided to the community by the GPs, and as healthcare delivery processes are usually initiated by them, they have a significant role in the overall volume of resources consumed in the health sector. There were about 86,000 GPs in Iran in 2019, accounting for more than 65% of all physicians in the country [2, 15]. Studies showed that over half of the GPs were working in the private sector, most of whom were self-employed and office-based [16]. Thus, this study aimed to evaluate the efficiency of GPs in Iran and the factors affecting it.

## Main text

### Methods

The present cross-sectional study made use of the data provided by Bayati et al. [17] in a survey in which a researcher-made tool and the convenient sampling method were used to collect the data of 666 GPs. The present study was restricted to self-employed physicians in the private sector.

In the present study, we examined self-employed GPs’ (n = 232) technical, managerial and scale efficiency using the Data Envelopment Analysis (DEA) method as well as the variable returns to scale assumptions and an output-oriented model (the goal of private GPs is usually product enhancement). General DEA model is a follows:

$$\begin{aligned} & {\text{Max}}h_{0} = \frac{{\mathop \sum \nolimits_{r = 1}^{s} u_{r} y_{r0} }}{{\mathop \sum \nolimits_{i = 1}^{m} v_{i} x_{i0} }}, \\ & {\text{Subject to}}:\frac{{\mathop \sum \nolimits_{r = 1}^{s} u_{r} y_{rj} }}{{\mathop \sum \nolimits_{i = 1}^{m} v_{i} x_{ij} }} \le 1;\,u_{r} ,\, v_{i} \ge 0; r = 1, \ldots , s ;i = 1, \ldots , m;j = 1, \ldots ,n \\ \end{aligned}$$

where y, x, u and v are output, input, output weight and input weight, respectively.

Based on previous studies [11–14] and the available data, physician’s working hours, number of staff, and space were considered as input variables, and the number of visits was considered as output in the present study. In this study, technical inefficiencies of the GPs were estimated. So efficiency could be given the score 1 (full efficiency) and more than one (inefficiency).

After estimating the GPs’ inefficiency scores, univariate analysis was performed to compare inefficiency scores based on demographic, practice and viewpoint factors.

After that, the factors affecting inefficiency were evaluated using the following regression model:

$$IE_{i} = \beta_{0} + \beta_{1} D_{i} + \beta_{2} P_{i} + \beta_{3} V_{i} + u_{i} i = 1,2, \ldots ,232$$

In which IE is the GPs’ technical inefficiency (dependent variable), D stands for demographic factors including gender, age, and marital status, P represents the variables related to medical practice including competition in the market, medical practice experience, location of practice, contract with insurers, and the existence of a pharmacy near the practice site, and V shows economic perspectives (i.e. GPs’ economic expectations). *u*_*i*_ represents the regression error, *i* shows the GPs, and *βs* is the coefficients of the model.

Competition in the market was measured by the number of centers offering similar services near the location of the physician’s practice (other physicians’ offices, clinics and hospitals). Economic expectations is a variable derived from three items, including economic expectations of the community, the physician’s family, and the physician him/herself [18]. The mentioned model was estimated using ordinary least square (OLS). F statistic was estimated to determine overall significance of model.

The Stata14.2 software was used for data analysis.

### Results

Table 1 shows the descriptive statistics of the demographic variables, the ones related their medical practice and the attitudes of the GPs studied.

**Table 1:** Descriptive statistics of studied self-employed GPs characteristics

Variables	Frequency	Percent
Gender		
Male	134	57.76
Female	98	42.24
Marital status		
Single	25	10.87
Married	205	89.13
Age (years)		
26–35	27	11.79
36–45	65	28.38
46–55	93	40.61
56 ≤	44	19.21
Practice experience (years)		
1–5	30	13.27
6–10	35	15.49
11–15	43	19.03
16–20	57	25.22
≤ 21	61	26.99
Practice location		
Tehran	90	44.12
Other province centers	52	25.49
Other cities and villages	62	30.39
Contract with the insurer		
No	80	37.74
Yes	132	62.26
Economic expectations		
Very low	39	17.49
Low	50	22.42
Moderate	4	1.79
High	84	37.67
Very high	46	20.63
Number of centers offering similar services near the location of the GP’s practice		
No	4	1.74
1–2	49	21.30
3–5	76	33.04
5 ≥	101	43.91
Existence of a pharmacy near the practice site		
No	4	1.73
Yes	227	98.27

About 2% of the GPs were fully efficient and the remaining (98%) were inefficient.

Based on the results, 2.09% of the physicians had constant returns to scale, and 31.41% and 66.49% of them had increasing and decreasing returns to scale, respectively.

The univariate analysis (Table 2) showed, the physicians’ inefficiency scores were significantly different based on gender, medical practice experience, location of practice, economic expectations, and contract with insurance organizations (P < 0.01).

**Table 2:** GPs’ inefficiency scores based on demographic, practice and viewpoint factors

Variables	Technical efficiency mean (CD)	P-value	Managerial efficiency mean (CD)	P-value	Scale efficiency mean (CD)	P-value
Overall	8.735 (11.253)		7.559 (10.565)		1.413 (2.450)	
Gender						
Male	6.791 (8.159)	0.002	6.117 (7.716)	0.014	1.153 (.298)	0.058
Female	11.94 (14.54)		9.942 (13.803)		1.844 (3.952)	
Marital status						
Single	11.598 (19.548)	0.249	10.618 (19.583)	0.188	1.328 (.741)	0.8712
Married	8.447 (9.995)		7.242 (9.105)		1.425 (2.579)	
Age (years)						
26–35	13.241 (13.572)	0.271	11.742 (13.731)	0.274	1.683 (2.538)	0.561
36–45	8.420 (7.574)		6.669 (5.378)		1.755 (4.316)	
46–55	8.188 (12.946)		7.236 (12.310)		1.207 (0.417)	
56≤	7.864 (9.966)		7.221 (9.837)		1.215 (0.561)	
Practice experience (years)						
1–5	11.208 (11.204)	0.000	9.880 (11.079)	0.001	1.632 (2.432)	0.144
6–10	17.387 (20.799)		14.031 (20.244)		2.434 (5.727)	
11–15	7.788 (8.709)		7.249 (8.624)		1.086 (0.101)	
16–20	6.572 (6.437)		5.619 (5.171)		1.211 (0.416)	
≤ 21	5.404 (4.248)		4.889 (4.105)		1.142 (0.231)	
Practice location						
Tehran	12.027 (15.966)	0.010	10.216 (15.206)	0.034	1.823 (4.006)	0.270
Other province center	7.806 (7.991)		6.697 (7.178)		1.228 (0.500)	
Other cities and villages	5.809 (5.599)		5.228 (5.355)		1.127 (0.200)	
Contract with the insurer						
No	15.344 (17.011)	0.000	12.984 (16.398)	0.000	1.917 (4.182)	0.060
Yes	5.432 (3.726)		4.836 (3.505)		1.166 (.355)	
Economic expectations						
Very low	5.648 (2.928)	0.002	4.854 (2.789)	0.0009	1.287 (0.707)	0.726
Low	8.378 (10.553)		6.981 (9.664)		1.747 (3.915)	
Moderate	28.647 (22.167)		27.536 (23.186)		1.186 (0.331)	
High	8.000 (8.819)		6.853 (7.280)		1.182 (0.326)	
Very high	10.662 (16.083)		9.685 (16.033)		1.183 (0.303)	
Number of centers offering similar services near the location of the GP’s practice						
No	5.715 (2.926)	0.098	4.334 (2.250)	0.063	1.362 (0.193)	0.453
1–2	8.635 (10.178)		7.116 (8.740)		1.933 (4.858)	
3–5	6.325 (5.260)		5.253 (4.080)		1.378 (1.524)	
≤ 5	10.938 (14.761)		9.854 (14.311)		1.176 (0.366)	
Existence of a pharmacy near the practice site						
No	6.142 (2.972)	0.688	5.136 (1.664)	0.690	1.159 (.182)	0.857
Yes	8.777 (11.334)		7.598 (10.644)		1.417 (2.470)	

According to the regression results (Table 3), gender had a significant relationship with inefficiency, and women were more inefficient than men. Age had also a positive relationship with inefficiency, and increased age would increase inefficiency. The experience of medical practice had a negative relationship with inefficiency, and its increase was followed by decreased inefficiency. The doctors who had contracts with insurance organizations were less inefficient. Furthermore, economic expectations had a positive relationship with inefficiency, and physicians who had higher economic expectations were more inefficient.

**Table 3:** Regression estimates for factors affecting technical inefficiency of Iranian self-employed GPs

Explanatory variable	Coefficient	P-value
Constant	− 6.925	0.521
Gender		
Male	Reference	
Female	3.776	0.072
Age (years)	0.475	0.013
Practice experience (years)	− .477	0.015
Practice location		
Tehran	Reference	
Other province centers	− 2.351	0.299
Other cities and villages	− 2.433	0.324
Contract with the insurer		
No	Reference	
Yes	− 6.475	0.005
Economic expectations	1.939	0.014
Competition in the market	1.710	0.127
Existence of a pharmacy near the practice site		
No	Reference	
Yes	− 1.734	0.794
Overall significance		
F statistic	5.42	0.000
R^2^	0.262	

### Discussion

The maximum scores of scale, managerial and technical inefficiency of the GPs evaluated in this study were 32.5, 88.103 and 88.425, respectively. There were wide inefficiency score changes. In addition, the scores indicated that the physicians were in a better situation in terms of scale efficiency than managerial efficiency. Scale efficiency indicated how close the physicians were to the optimal scale of production (constant returns to scale). Given the status of returns to scale, most of the physicians needed to change (increase or decrease) their visitation rate to provide services at an optimum production scale. About 2% of the GPs were fully efficient technically and the remaining 98% were inefficient. This showed that they did not use their resources properly (time, physical and human resources).

A study carried out by Olsen et al. [12] in 2011 on GPs in Denmark showed that the mean technical efficiency was 0.79 to 0.84. Similar to the current study, the range of efficiency scores was wide and many GPs were inefficient. However, their findings somewhat differ from those of the present study, the reason for which could be different outputs, inputs, sample sizes, and socioeconomic conditions. In the Olsen study the stochastic frontier analysis was used, the inputs were the physicians’ and nurses’ working hours and the outputs were the number of visits and the total productivity index. The sample size was 1749 as well.

According to the findings of the present study, gender, age, medical practice experience, contract with insurance companies, and economic expectations had a significant relationship with GPs’ inefficiency.

Women were more inefficient than men (at 10 percent significance level). Only a similar study reported a related finding; the study by Olsen et al. [12] on Danish GPs showed that women were more inefficient than men when considering the number of visits as an output. It seems that due to women’s different preferences, expectations and financial responsibility compared to men [19, 20], they pay less attention to the economic management and efficiency of their practices. A study showed that male GPs in Iran worked approximately 50 h more than women per month and had about 80 more visits [17]. Male physicians had also higher earnings expectations than female ones [9]. In other studies carried out in other countries, such as France and Australia, female GPs were also less productive than males [21, 22].

The results showed that GP’ inefficiency increased with age. A similar study by Olsen et al. [12] showed that age had a significant relationship with inefficiency of GPs. Another study showed that GPs in Iran worked longer hours as their ages increased, but had fewer visits [17]. Doctors themselves choose how long to work and whether to have leisure time or to visit patients. In other words, economic behavior of physicians varies according to their utility function. Besides, based on the target income hypothesis, differences in their financial expectations can lead to differences in their performance [23]. Older physicians may have reached their desired financial levels and reduce their activities. On the other hand, studies outside the health sector have noted that as people age, their willingness and also ability to work (especially in hard jobs) decrease [24, 25].

The results showed that an increase in medical practice experience would lead to decreased inefficiency of GPs. Trust are the most important element of the physician–patient relationship [26]. Patients are more trusted in experienced physicians and refer to them; thus, more experienced physicians have higher visit production efficiency. Besides, as more experienced physicians have higher financial expectations [9, 27], they are likely to produce more with available resources.

One important point is that physicians’ inefficiency was negatively correlated with the practice experience and positively with age. In the present study, since the variables of age and practice experience were both included in the regression model, their effects could be interpreted purely. This indicates that more experienced physicians are not necessarily older.

Having a contract with insurance company had a negative relationship with inefficiency. In other words, doctors who had a contract with an insurer were less inefficient. Since the patients having an insurance look for doctors who have insurance contract to be able to use the insurance coverage advantages, there is a higher demand for physicians who are contracted with insurance organizations. The positive impact of insurance coverage on the demand for health services have been confirmed in various studies [28, 29]. Therefore, in equal conditions, physicians who have insurance contracts have more visits and are more efficient.

The results also showed that general practitioners with higher economic expectations were more inefficient. This suggests that when other conditions (gender, age, work experience, insurance contract, competition, and location of practice) were controlled, higher economic expectations would not necessarily mean more optimal visit production, and on the contrary, such physicians were less efficient.

## Limitations

The present study had some limitations. First, since there was no accurate sampling frame and address list for the GPs in the private sector, the convenience sampling method was used, which might reduce the generalizability of the results. The second limitation was the relativity of the efficiency concept. Efficiency estimates depends on the number and type of inputs and outputs, sample size, model assumptions (input- driven or output-driven), and model estimation method. If these items change, efficiency score will change, too. Therefore, the findings should be interpreted with caution. The third limitation was the lack of a complete output criterion for the physicians’ performance. The output criterion in this study, like in many other studies, was visits. Visits could not show the quality of services and the difference between the services provided by physicians. The last limitation is that because of data unavailability, we could not assess the effect of patient’s preferences on GP’s efficiency. Patient’s characteristics such as their preferences, severity of disease, socio-demographic features and economic status can affect demand for GPs visits and so their efficiency. In this regards, only one research considered patient characteristic as a compound variable from some demographic and socio-economic factors [12].

## Data Availability

The data used in this study are not publicly available because the participants were promised that the raw data would remain confidential. However they are available from the corresponding author on reasonable request.

## Abbreviations

DEA: Data envelopment analysis
GP: General practitioner
OLS: Ordinary least square
